# Casein and Casein-Derived Peptides: Antibacterial Activities and Applications in Health and Food Systems

**DOI:** 10.3390/nu17101615

**Published:** 2025-05-08

**Authors:** Tomás Moita, Laurentina Pedroso, Isabel Santos, Ana Lima

**Affiliations:** 1 Research in Veterinary Medicine (I-MVET), Faculty of Veterinary Medicine, Lusófona University, Lisbon University Centre, Campo Grande, 376, 1749-024 Lisbon, Portugal; tgmoita@gmail.com (T.M.); laurentina.pedroso@ulusofona.pt (L.P.); maria.isabel.santos@ulusofona.pt (I.S.); 2 Veterinary and Animal Research Centre (CECAV), Faculty of Veterinary Medicine, Lusófona University, Lisbon University Centre, 1749-024 Lisbon, Portugal; 3 IPLUSO—Polytechnic Institute of Lusofonia, School of Health, Protection and Animal Welfare, Campo Grande 400, 1700-098 Lisbon, Portugal

**Keywords:** milk, casein, casein-derived peptides, antibacterial activity, food bioactives, functional foods, clinical applications

## Abstract

The growing threat of antimicrobial resistance has intensified the search for alternative strategies to conventional antibiotics and preservatives. Casein-derived antimicrobial peptides (CDAMPs), generated through proteolysis, exhibit potent activity against a broad spectrum of pathogens, including antibiotic-resistant strains, revealing strong potential as natural preservatives and therapeutic agents in food and medical applications. Furthermore, casein can be an ideal source for peptide production in these sectors due to its abundance, disordered structure, which enhances enzymatic cleavage, and its amino acid profile, which favors bioactivity. Nonetheless, there is limited literature addressing real-life applications in veterinary medicine, food safety, and public health. This review provides a structured synthesis of current knowledge on the antibacterial properties of CDPs. We classify the main types of these peptides, describe their production methods, and summarize their mechanisms of action against Gram-positive and Gram-negative bacteria. Furthermore, we examine their potential applications in clinical, veterinary, and food-related contexts, and discuss key aspects related to delivery systems, safety, and regulatory considerations. Overall, our findings highlight the potential of CDPs in addressing antimicrobial resistance, reducing antibiotic use in livestock and humans, and contributing to sustainable food safety and functional food production.

## 1. Introduction

Caseins are the predominant proteins in milk and have long been valued for their nutritional and functional properties. Etymologically derived from the Latin word *caseus* (cheese), the term casein reflects its historical use in cheese production [1]. While dairy products rich in casein have long been associated with therapeutic benefits, including enhanced satiety, improved nutritional value, and muscle maintenance [2,3,4,5], caseins and their derivatives have gained considerable attention in the food industry as stabilizers and carriers in complex formulations such as emulsions, foams, and nanoparticles [6,7]. Nonetheless, one of the most notable and promising characteristics of caseins and their derivatives is their ability to release bioactive peptides through enzymatic hydrolysis, microbial fermentation, or gastrointestinal digestion [2,8,9,10]. These encrypted peptides, which are inactive within the native protein structure, can exert a wide range of physiological effects once liberated and absorbed. Documented bioactivities include antihypertensive, immunomodulatory, antioxidant, and antimicrobial effects [9]. Among these, antimicrobial activity has gained increasing attention due to its potential in addressing the global antimicrobial resistance (AMR) crisis [11,12]. An expanding body of evidence demonstrates that specific casein-derived peptides (CDPs) inhibit various pathogenic bacteria via diverse mechanisms that may bypass conventional resistance pathways [13,14]. Due to their natural origin, biocompatibility, and low toxicity, their application can be safely extended to functional foods, nutraceuticals, and veterinary products [10], with potential relevance for human and animal health, food preservation, and clinical nutrition [15,16]. Being the most abundant milk proteins, caseins offer a sustainable and accessible source for peptide production and are particularly well suited for the generation of bioactive peptides due to their intrinsic structural characteristics. On the one hand, unlike globular proteins, caseins are classified as intrinsically disordered proteins (IDPs), which lack a rigid tertiary structure. This structural disorder enhances their susceptibility to enzymatic cleavage during digestion, fermentation, or processing, facilitating the release of functional peptides [17]. Furthermore, caseins form micellar structures—colloidal aggregates that improve solubility and allow slow, sustained release of peptides under physiological conditions [18,19]. These micelles contribute to prolonged peptide bioavailability, which is particularly advantageous for therapeutic and functional food applications. Additionally, caseins are enriched in hydrophobic and cationic amino acids such as lysine, arginine, and proline—residues commonly found in antimicrobial peptides, which confer amphipathic properties that allow interaction with bacterial membranes and may also play roles in mineral binding [20,21,22,23].

Nevertheless, not unlike for other milk-derived peptides, a clear gap remains in the translation of CDPs into applied contexts. There is limited literature addressing aspects such as delivery systems, regulatory classification, or real-life applications in veterinary medicine, food safety, or public health. This work aims not only to consolidate existing knowledge on the antimicrobial properties of CDPs, but also to assess their practical relevance, implementation strategies, and potential for integration into clinical, veterinary, and food systems. By bridging basic and applied research, this review aims to inform the development of effective, safe, and sustainable antimicrobial strategies based on casein bioactive peptides.

This review includes studies involving caseins and casein-derived peptides from bovine, ovine, and caprine milk. However, due to the heterogeneity of sources in the literature and the general focus of this review on casein structure and bioactivity, the specific animal origin of each protein or peptide is not systematically identified throughout the text. Where the source species was explicitly stated in the original study and relevant to the interpretation, it is mentioned. Nonetheless, the overall discussion emphasizes general casein characteristics and functionalities, regardless of species-specific variation.

## 2. Caseins’ Structure

Milk consists primarily of water (85–87%), fats (3.8–5.5%), proteins (2.9–3.5%), and carbohydrates (5%) at the macroscopic level [24]. At the microscopic level, it contains vitamins, minerals, organic acids, biogenic amines, nucleotides, immunoglobulins, and oligosaccharides [24,25]. Of the total protein content, approximately 80% is casein, with the remaining 20% being whey proteins [8,22].

Caseins are encoded by single autosomal genes [1]. The major casein proteins include αS1-casein (CSN1S1), αS2-casein (CSN1S2), β-casein (CSN2), and κ-casein (CSN3), which represent approximately 40%, 12.5%, 35%, and 12.5% of the total casein fraction, respectively [1,22]. Comparative genomic studies across species, including cows, humans, camels, and elephants, show that CSN1S1 and CSN2 are closely related, while CSN3 is the most divergent, with CSN1S2 occupying an intermediate position [1]. Genetic polymorphisms lead to significant heterogeneity among casein proteins, producing various protein variants. These variations arise from point mutations, peptide fragment deletions, and post-translational modifications such as glycosylation, phosphorylation, or partial hydrolysis [22].

Structurally, caseins exhibit low homology in their primary sequence due to extensive genetic variability. Their secondary structure is characterized by a paucity of α-helices and β-sheets, conferring flexibility and a random coil conformation. This plasticity enables intermolecular electrostatic, hydrogen, and hydrophobic interactions. The tertiary structure is poorly defined, while the quaternary structure consists of micellar aggregates [20,22].

### 2.1. Casein Micelles

Caseins form micelles, colloidal aggregates that are essential for the stability and bioavailability of milk proteins. These micelles consist of approximately 95% protein and 5% minerals, primarily calcium phosphate (MCP), which is composed of calcium, phosphate, magnesium, and citrate [26,27]. The structure of casein micelles is not fully understood, but they are described as nearly spherical with a radius of 50–150 nm [1].

The κ-casein molecules are predominantly located on the surface of the micelles, stabilizing the structure through electrostatic repulsion and steric hindrance. This stabilization is influenced by the length and density of the κ-casein glycoprotein brushes. When the κ-casein brushes collapse, as in acidification or ethanol treatments, casein molecules aggregate, leading to gel formation [27].

### 2.2. Functional Properties

Caseins exhibit advantageous nutritional and technological properties, attributed to their stability under processing conditions, protection against oxidative damage, and high bioavailability. Functionally, they play a central role in milk coagulation, a fundamental step in cheese production. Coagulation, whether induced by acid or rennet, initiates upon the neutralization of the negative charges on κ-casein, ultimately leading to casein precipitation and gel formation [1]. Beyond this, caseins possess molecular chaperone activity, acting to stabilize other proteins under thermal or physiological stress by preventing their aggregation and denaturation—an attribute of particular relevance during processes such as milk pasteurization [1].

Among the casein fractions, αS1-casein is the most abundant, with a molecular mass of 23.6 kDa and comprising 199 amino acids, 8.4% of which are proline residues [22,27]. Four genetic variants (A, B, C, and D) are described, with variant B being the most prevalent. Structurally, αS1-casein is characterized by a disordered conformation, with a limited presence of α-helices and β-sheets [17,28]. Similarly, αS2-caseins possess a molecular weight ranging from 25.2 to 25.4 kDa, composed of 207 amino acids, and share comparable secondary structural features [22,27].

Both αS1- and αS2-caseins have been shown to exert chaperone-like activity [6,22]. This property is typically evaluated in vitro by subjecting a target protein to defined stress conditions (e.g., thermal, oxidative, or reductive), in the presence of varying concentrations of the putative chaperone [6]. In the milk matrix, αS1-caseins are critical for the stabilization of other proteins, such as β-casein and β-lactoglobulin, preventing their denaturation and aggregation [22]. The chaperone activity of αS1- and αS2-caseins has been reported to resemble that of small heat shock proteins (sHsps) and clusterin. sHsps, with molecular weights in the range 15–30 kDa, differ from classical heat shock proteins in that they do not require ATP for function. They bind to misfolded or stressed proteins, stabilizing them through the formation of large molecular complexes that hinder aggregation [6].

β-casein, with a molecular weight of approximately 24 kDa and 209 amino acids [22,27], contains secondary structural motifs such as β-sheets and β-turns and is considered the most hydrophobic of all caseins. To date, 12 genetic variants of β-casein have been identified (A1, A2, A3, B, C, D, E, F, G, H1, H2, and I) [22,27,29], with particular attention given to variants A1 and A2 due to their potential nutritional and technological implications [30,31]. As an amphiphilic protein, β-casein has the ability to self-assemble into micellar structures under physiological or acidic conditions, orienting its hydrophobic domains toward the micellar core while exposing hydrophilic regions to the external environment [22]. Moreover, β-casein displays a reversible tendency to dissociate from micelles at low temperatures, a property that significantly affects micellar integrity during cold storage or isolation procedures [32,33].

κ-casein, distinct from α- and β-caseins, is a glycoprotein composed of 169 amino acids and a molecular mass of approximately 19 kDa. Its various isoforms differ in their degree of glycosylation, contributing to functional diversity in milk. To date, 11 genetic variants of κ-casein have been described [22,27].

## 3. Bioactive Peptides Derived from Caseins

**Figure 1:**
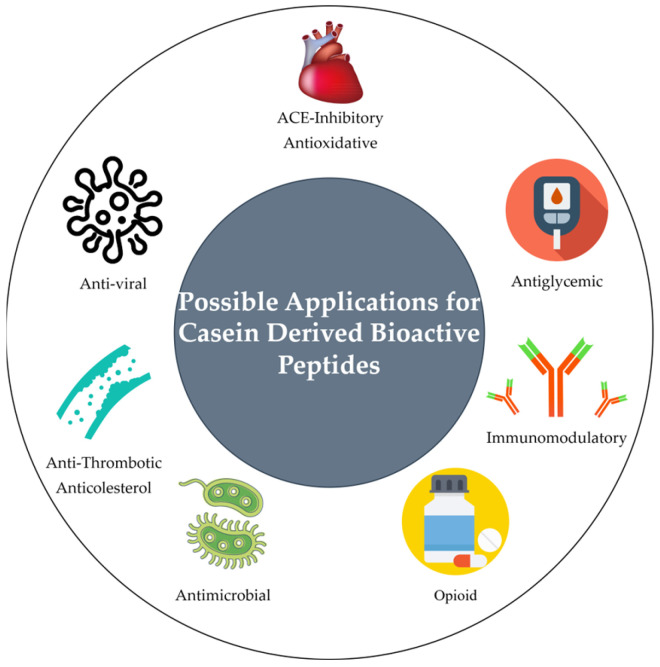
Different possible applications for casein-derived bioactive peptides. Adapted from [36,37].

Beyond their well-recognized nutritional role, caseins constitute a reservoir of encrypted bioactive peptides with significant biological potential [1,27]. These casein-derived peptides (CDPs) have garnered increasing scientific interest due to their wide spectrum of bioactivities, which may confer beneficial effects on both human and animal health [10]. Numerous physiological functions have been attributed to these peptides, including antimicrobial, antihypertensive, opioid-like, and immunomodulatory actions [34]. The diversity of these biological effects arises from specific amino acid sequences within the parent casein proteins that, upon enzymatic release, can interact with distinct molecular targets in the organism [35]. Figure 1 illustrates the principal bioactivities and potential applications associated with bioactive peptides derived from casein.

In terms of cardiovascular health, some CDPs, like casokinins, exhibit angiotensin-converting enzyme (ACE)-inhibitory effects, which help regulate blood pressure [38]. This mechanism is similar to certain antihypertensive drugs but offers a natural, food-based intervention. With lifestyle-related hypertension on the rise, CDPs offer a potential dietary approach to managing blood pressure. Additionally, CDPs such as β-casomorphins interact with opioid receptors, resulting in mild analgesic and calming effects [39]. These effects may be beneficial for stress management, while the peptides’ ability to modulate immune responses and exert anti-inflammatory effects could play a role in managing immune-related diseases [38,40]. Some of the most known are described in Table 1. This broad range of effects underscores the importance of CDPs in the development of functional foods targeting mental wellness and immune support.

### 3.1. Production of CDPs

**Figure 2:**
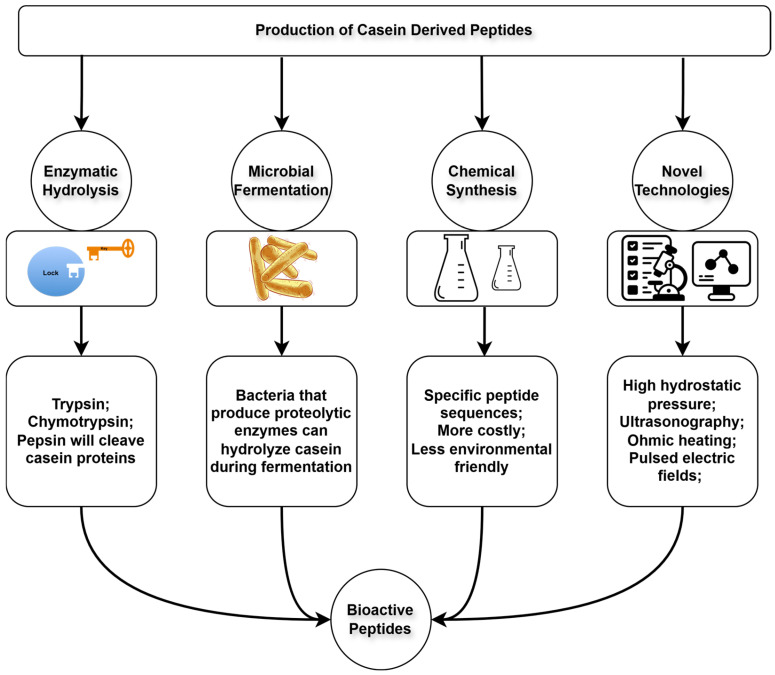
Main production methods to obtain bioactive peptides adapted from [10,15,51,53].

There are multiple methods for the production of casein-derived antimicrobial peptides [36,49] (Figure 2), although certain requirements must be met by the peptide, namely minimizing undesirable side effects in the organism and exhibiting minimal immunoreactivity [37,50].

The main methodologies include enzymatic production and microbial fermentation [10,16,51], although there are several other emerging methods [52].

The most common strategies for producing milk-derived peptides are microbial proteolytic activity, extracellular proteases, food-grade enzymes, and the application of recombinant DNA technology [23], with the fermentation route by LAB being the most common [8]. Enzymatic hydrolysis, widely used in the food and pharmaceutical industries [54], offers advantages, including the ability to scale production with reduced reaction times and less residual solvent presence [10,16]. In vivo digestive enzymes such as pepsin, trypsin, and chymotrypsin are responsible for protein hydrolysis [55]. In vitro models simulate gastrointestinal digestion via proteolytic enzymes, including alcalase and thermolysin, in combination with pepsin and trypsin. Microbial fermentation, using lactic acid bacteria (LAB), has the advantage of lower costs, as microorganisms are less expensive than commercial enzymes [54]. This fermentation primarily uses bacteria or yeasts to hydrolyze proteins during their growth, with temperature and pH being a critical parameter in this method [10]. Starter cultures, composed of LAB consortia, ensure better control and consistency in the organoleptic characteristics of the final products [51,56], including *Lactococcus lactis*, *Lactobacillus helveticus*, and *Lactobacillus delbrueckii* ssp. *bulgaricus*. These cultures optimize and ensure greater control over fermentation processes used to obtain peptides with consistent organoleptic characteristics [51,56]. LAB are widely used in the food industry, particularly in the production of yogurt and cheese [57], and perform either homofermentative or heterofermentative fermentations, with the main difference being the byproducts generated, such as lactic acid, and in the case of heterofermentation, acetic acid, carbon dioxide, and ethanol [58]. One of the main advantages of using microorganisms for peptide production is that the lactic acid produced can acidify the medium and inhibit the growth of undesirable microorganisms [56,59]. Finally, the combination of proteolysis with high hydrostatic pressures has the benefit of increasing enzymatic activity without the need for prior thermal treatments, which reduces production costs and limits microbial growth, benefiting both the food and pharmaceutical industries [52].

Additionally, novel technologies have emerged to enhance the efficiency of bioactive peptide production, such as high hydrostatic pressure, ultrasonography, microwave-assisted extraction, ohmic heating, pulsed electric fields, and subcritical water hydrolysis [10,15,51,52,53,60]. Currently, these advanced technologies are often integrated with enzymatic hydrolysis or microbial fermentation to reduce production costs while enhancing peptide yield, bioactivity, and efficiency [61,62]. Peptidomics has also become an essential tool in dairy research, extending beyond the study of protein digestion. In the dairy industry, these tools are employed to identify peptides in various dairy products, such as kefir [63], yogurt [64], and cheeses [65,66,67,68] and can be used to isolate and track these targeted peptides throughout complex proteolytic processes [69].

### 3.2. Antibacterial Activities of CDPs

Overall, antibacterial activities in casein and its derived peptides have received significant attention due to their broad-spectrum antimicrobial properties and their potential application in food safety and health. So far, several dozens of antimicrobial peptides (AMPs) derived from milk have been extensively characterized. While some were named [8,70], numerous other bioactive sequences have been identified but remain unnamed. Overall, CDPs originate from different casein fractions, including α-S1-, α-S2-, β-, and κ-caseins while, for example, casecidin A and B are derived from α-casein, lactenin and isracidin from β-casein, and kappacin from κ-casein [70], to name a few of the most referenced. Most milk-derived peptides possess low molecular weights, generally below 10 kDa, which facilitates rapid interaction with microbial targets and may enhance their antimicrobial efficacy compared to peptides from other food sources [71], demonstrating both bacteriostatic and bactericidal activity against a wide range of pathogens, including antibiotic-resistant strains [70,72]. For example, CAMP211-225, identified in 2020, exhibits significant antibacterial activity against *Escherichia coli* and *Yersinia enterocolitica* [73]. This peptide was found in higher concentrations in preterm human milk and displayed minimum inhibitory concentrations (MICs) of 3.125 μg/mL and 6.25 μg/mL, respectively, against *E. coli* and *Y. enterocolitica*, acting mostly thought membrane disruption, contributing to rapid bacterial cell death [74].

To consolidate this growing body of knowledge, several online databases are now available that catalogue milk-derived peptides and their associated biological activities, with a particular emphasis on antimicrobial potential [31,37]. Table 2 describes the main CDPs and their potential bacterial targets, as well as their amino acid sequence and names.

Considering their origin, casein-derived antimicrobial peptides (CDAMPs) target a broad range of bacterial pathogens [59]. From the α-S1-casein fraction, several peptides have been identified. For example, isracidin inhibits *E. coli* DH5a, *E. coli* DPC6053, *Cronobacter sakazakii*, and others. A fragment of isracidin also shows activity against *E. coli* and *B. subtilis* [80,81]. Caseicin A, derived from α-S1-casein, targets *E. coli*, *C. sakazakii*, *L. innocua*, *L. bulgaricus*, and *S. mutans*. Similarly, caseicin B and caseicin C also inhibit *E. coli*, *C. sakazakii*, and *L. innocua*, among others [71,76,77,78]. Additionally, peptides such as CP1, derived from α-S1-casein, show antibacterial activity against *B. subtilis*, *L. monocytogenes*, *S.* Enteritidis, *S.* Typhimurium, and other strains [9,83,84]. These peptides are particularly noted for their broad-spectrum activity, which includes both Gram-positive and Gram-negative bacteria.

The α-S2-CDP group, including various fragments like casocidin, also exhibit notable antibacterial effects. Casein F targets pathogens such as *E. coli*, *S. carnosus*, *B. subtilis*, and *L. monocytogenes* [72,87,88,89,90]. Other peptides derived from this fraction, like CR1, CR3, and CR5/6, show inhibitory effects against a range of bacteria including *L. innocua*, *B. cereus*, *S. aureus*, and *S. Typhimurium* [46,93]. Additionally, p10 and p14 peptides from α-S2-casein have activity against *B. cereus*, *S. aureus*, *L. monocytogenes*, and *H. pylori* [91].

From the β-casein fraction, peptides like casecidin 17 and others such as Bc3, Bc6, and Bc8 show significant antimicrobial activity against *E. coli* [96]. These peptides have also been reported to inhibit *B. cereus*, *L. monocytogenes*, and *B. thuringiensis*. The β-CDP group primarily targets *E. coli*, demonstrating its role in combating enteric infections [81,85,97].

Lastly, peptides derived from κ-casein exhibit antimicrobial properties against a variety of pathogens including *E. coli*, *S. marcescens*, *L. innocua*, and *S. carnosus*. Some of the κ-CDP group also target *S. aureus*, *L. casei*, and *L. acidophilus*, making them effective against both Gram-positive and Gram-negative bacteria [85,97,103,104].

Overall, the CDPs listed in Table 2 shows promising antibacterial properties against a wide spectrum of pathogens, indicating their potential for use in food safety and therapeutic applications. As we can see, CDPs have shown effectiveness against both Gram-positive and Gram-negative bacteria, including common foodborne pathogens, namely *Salmonella* spp., that are a leading cause of foodborne illness worldwide, affecting millions each year in the U.S.A. and Europe [106]. *Listeria monocytogenes*, the agent responsible for listeriosis that is a rare but serious disease with a high fatality rate (20–30%), remains a significant public health concern, particularly in ready-to-eat foods [107]. Shiga toxin-producing *Escherichia coli* (STEC) is also responsible for severe foodborne outbreaks [108]. Although less serious, *Staphylococcus aureus* can cause food poisoning when enterotoxins are produced in improperly handled or stored foods [109].

These peptides’ ability to target both Gram-negative and Gram-positive bacteria highlights their versatility and potential as natural antimicrobial agents. Overall, α-S1-CDPs are the most studied. These peptides are noted for their broad-spectrum antibacterial activity, targeting a wide range of bacterial strains [71,76,77,78,79,81].

As for the bacteria, *E. coli* is the most frequently mentioned in the studies, and it is the target for many of the peptides derived from α-S1-casein, α-S2-casein, β-casein, and κ-casein. *E. coli* is particularly prevalent in the list, with various strains such as *E. coli* DH5a, *E. coli* NEB 5α, *E. coli* ATCC 25922, and others being commonly referenced as targets for these peptides [9,75,79,81,85,88,97,104]. This suggests that many of these CDPs are effective against *E. coli*, a major pathogen in both human and animal health, particularly in gastrointestinal infections. [74,110].

In a more recent study by Shizhe Qi and his colleagues, casein-derived antimicrobial peptide mixtures were generated via optimized proteolysis, showing strong inhibitory effects against *Streptococcus mutans* and *Porphyromonas gingivalis*, including disruption of bacterial membranes and biofilm formation. Proteomic analysis identified 301 potential CDPs, with four novel peptides confirmed synthetically [111].

#### In Silico Prediction and Peptidomic Characterization of CDAMPs

In recent years, in silico methodologies have emerged as powerful tools in the identification and characterization of casein-derived AMPs, offering a complementary and time-efficient alternative to traditional biochemical approaches. These strategies accelerate the discovery pipeline by enabling rapid screening of peptide sequences, prediction of bioactivity, and assessment of safety profiles. For instance, a study employing the BIOPEP-UWM platform simulated gastrointestinal digestion of goat milk proteins, identifying 83 potential AMPs, with β-casein contributing the highest number of candidates [112]. These peptides were subsequently evaluated using machine learning algorithms to predict potential antimicrobial activity, along with assessments of potential physicochemical properties, toxicity and allergenicity by analyzing their amino acid sequences and predicting their likelihood of eliciting an immune response [112]. From this dataset, peptide sequences such as YLEQLLR and VLNENLLR derived from β-casein were computationally predicted to exhibit antimicrobial activity against *Listeria monocytogenes*, *Escherichia coli*, and *Salmonella enterica*, and these predictions were later validated through in vitro assays [112]. A related investigation extended this approach to the proteomes of goat and sheep milk, as well as feta cheese, employing an in silico workflow to predict and characterize AMP candidates. Peptides were ranked based on antimicrobial potential and stability against gastrointestinal proteases, providing insight into their likely functionality in physiological conditions [113]. In parallel, recent advances in mass spectrometry-based peptidomics have substantially enhanced the empirical identification of bioactive casein peptides. In a 2017 study, the peptide composition of casein hydrolysates generated under various enzymatic hydrolysis conditions was investigated through mass spectrometric analysis [114]. The authors identified a total of 70 unique peptides, including 28 from αs1-casein, 21 from αs2-casein, 13 from β-casein, and 8 from κ-casein. Most peptides had molecular weights below 5000 Da, with the majority falling in the range 1000–1500 Da. All peptides were predicted to be non-toxic and non-allergenic, and several exhibited high bioactivity scores as determined by PeptideRanker analysis [114].

### 3.3. Mechanisms of Action of CDPs

**Figure 3:**
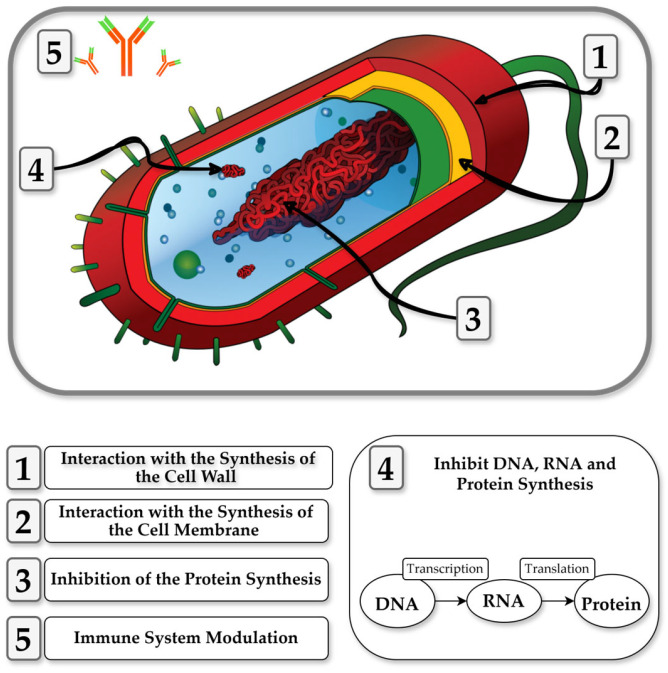
Proposed mechanisms of action of casein-derived antibacterial peptides, including membrane disruption, pore formation, inhibition of intracellular targets (e.g., DNA/RNA synthesis), and interference with metabolic pathways. Adapted from [8,14,46].

Milk-derived peptides exhibit diverse antibacterial mechanisms and have been extensively studied [23]. Their antimicrobial activity arises from several key actions, including bacterial membrane disruption, inhibition of cell wall synthesis, and interference with bacterial metabolism [23]. Structural features such as amino acid composition, hydrophobicity, and net charge play a critical role in determining their bioactivity [115,116,117]. It has been proposed that these peptides act through multiple simultaneous mechanisms, and a group target model has been suggested to describe this complex mechanism of action [115,116,117]. Figure 3 depicts the main mechanisms of CDAMPs.

CDAMPs’ antimicrobial properties are primarily due to their cationic and amphipathic nature. Their positive charge enables interactions with the negatively charged bacterial membranes, where they can insert into the lipid bilayer, disrupting membrane integrity and increasing permeability. This results in the leakage of essential ions and nutrients, leading to bacterial cell death [116,118]. CDPs are effective against both Gram-positive and Gram-negative bacteria, including *Escherichia coli* and *Staphylococcus aureus* [53,118]. Casecidi acts by forming pores in the bacterial membrane, causing uncontrolled leakage of cellular contents and ultimately leading to cell death [53]. In contrast, peptides like αS1-CDP destabilize the membrane by thinning the lipid bilayer, further compromising its structural integrity [119,120]. Some CDPs also disrupt bacterial membranes by destabilizing the electrochemical gradient and hindering ATP production via ion channel interference [14]. Beyond membrane disruption, some CDPs interfere with intracellular processes, such as DNA and RNA synthesis, as β-CDPs which have been shown to interact with dihydrofolate reductase and DNA gyrase in *S. aureus*, impairing bacterial replication [121]. β-casein-derived peptides have also been shown to interact with key enzymes like dihydrofolate reductase and DNA gyrase, impairing bacterial replication and contributing to their antimicrobial efficacy [121]. Additionally, CDPs can also generate reactive oxygen species (ROS), contributing to further damage of bacterial cells and enhancing their bactericidal effects [122].

Notably, several CDAMPs appear to exert antibacterial effects primarily against pathogenic strains, with minimal or no reported activity against beneficial dairy-associated bacteria. This selective behavior is likely related to differences in membrane composition and electrostatic properties and may reflect a broader trend observed in other milk-derived peptides. Their cationic charge facilitates binding to negatively charged membranes, while their amphipathic structure allows them to penetrate the lipid bilayer, disrupting membrane integrity and increasing permeability [115,118]. Additionally, some peptides may have specific features that target specific pathogens: GMp7 (KVLPVPQ), a milk-derived antimicrobial peptide described in 2025, was shown to be highly effective against *S. aureus* due to specific structural features that contributed to its selectivity [123]. Also, certain peptides, such as αs165-181, have been found to bind to specific pathogenic genomic DNA [124]. In summary, CDPs are versatile antimicrobial agents that function through multiple mechanisms, but require further investigation into their effects on beneficial microbiota and potential applications in clinical settings.

It is noteworthy that, besides CDPs’ antibacterial activity, several studies have explored the antifungal activity of CDPs, although this area of research is less explored. One relevant study showed that casein protein hydrolysates from cows’ milk exhibited antifungal activity against *Candida krusei*, with inhibition rates varying depending on the type of hydrolysis and the peptide concentration. These hydrolysates showed effectiveness not only against *Candida* species but also against other pathogenic fungal species, suggesting that casein could be a valuable source for the development of natural antimicrobial compounds [125]. Furthermore, peptides derived from goat milk casein, when hydrolyzed by enzymes such as trypsin, also exhibited antifungal effects against species like *Candida albicans*. [126]. These studies indicate that CDPs, in addition to their antimicrobial properties, also hold potential as antifungal agents, providing a natural and effective alternative for the control of fungal infections, both in medicine and in the food industry, as natural preservatives.

## 4. Practical Applications of CDPs

**Figure 4:**
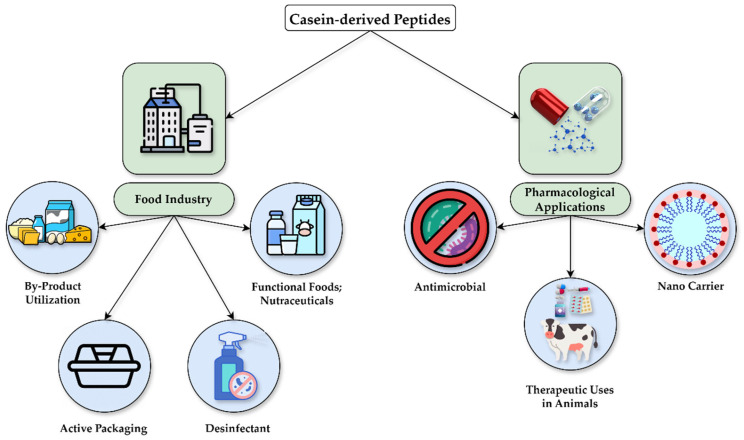
Possible practical applications of casein-derived peptides in food and clinical systems.

Caseins and CDPs present several features that render them good candidates for several applications for both food and health settings. On the one hand, casein’s well-known ability to form micelles greatly enhances its potential as a carrier for biologically active agents. This property not only increases the stability of encapsulated compounds but also improves their bioavailability, further making casein an excellent candidate for the delivery of bioactive molecules, including drugs, dietary supplements, and functional food ingredients, which has been broadly researched [20]. Another feature is casein and CDPs’ ability to combine with metal ions, allowing the formation of metal–protein complexes with interesting bioactivities. Qu et al. used casein peptides derived from dairy waste to capture Cu^2+^ from industrial wastewater, forming CPs/Cu/Cu_2_O nanoparticles, which in turn exhibited strong antibacterial activity against *E. coli*, *S. aureus*, and multidrug-resistant strains from livestock wastewater by generating reactive oxygen species and releasing Cu^2+^ [127]. Additionally, CDAMPs have become increasingly significant due to their promising applications as functional ingredients and nutraceuticals. While their natural origin appeals to both consumers and producers looking for safer, more sustainable alternatives to synthetic preservatives [128,129], several studies indicate CDPs are non-toxic and non-allergenic, further supporting their use in both food and pharmaceutical products [114]. Corroborating this is a growing body of evidence that focuses on improving the production processes of these peptides, as well as finding novel methods to stabilize them in different food and health-related products. Overall, the possible practical applications of CDAMPs are summarized in Figure 4 and comprise several areas of interest in both food and health systems. These are described in further detail below.

### 4.1. Application in the Food Industry

The global market for milk-based products and ingredients is expanding steadily, being valued at USD 13.67 billion in 2024 and expected to grow at a compound annual growth rate (CAGR) of 6.3% from 2025 to 2030. A key driver of this expansion is the increasing demand for protein-fortified foods and beverages, particularly those enriched with milk protein hydrolysates, due to their enhanced digestibility and functional properties. This trend is especially significant in developed regions such as Europe and North America, where health-conscious consumers are increasingly opting for convenient, nutrient-dense options such as functional yogurts, protein bars, and ready-to-drink formulations [130]. Whey protein concentrate (WPC) and whey protein isolate (WPI) are widely used in the protein ingredient market due to their cost-effectiveness, solubility, and emulsification capacity [131]. However, casein and casein bioactive peptides derived from casein are increasingly appreciated for their distinctive bioactivity and functionality [37,132]. Today, casein’s mineral absorption and binding properties are extensively explored in industrial contexts, especially in the development of beverages such as sports drinks designed for mineral supplementation, as well as in clinical nutrition for disease management [37]. CDPs such as casomorphins and isracidin are incorporated into functional foods and nutraceuticals due to their ability to exert antioxidant, antimicrobial, and antihypertensive effects [37,132]. CDPs also show promise as ingredients in hypoallergenic products, such as infant formulas, where they can contribute to immunomodulation and reduce allergic responses [76]. Additionally, they help in enhancing the sensory attributes of foods by providing improved texture and stability. In dairy processing, fortification with micellar casein enhances the rheological and functional characteristics of yogurt [133] and β-casein has been shown to prevent the degradation of anthocyanins such as those found in blueberries [134]. Additionally, functional properties of casein can be improved through glycation with polysaccharides, which enhances emulsifying and foaming behavior, making it suitable for applications such as emulsifiers and for encapsulating bioactive molecules [135].

#### 4.1.1. Food Preservation and Safety

Several reports substantiate that CDPs can be natural alternatives to synthetic preservatives, which are often criticized for potential health risks [136,137,138,139,140]. Kappacin and isracidin have been incorporated into dairy products, meats, and other perishable foods, showing promise in different food matrices [82,136]. Although some reports suggest peptide degradation remains a concern in food matrices, limiting its action time [136], casein-derived casocidin and isracidin [137] have shown resistance to microbial proteolysis, especially in yogurt starter strains, which supports their use as preservatives at low pH. BCp12 maintains antimicrobial activity under various salt concentrations, although it remains sensitive to high temperatures and enzymatic degradation [138]. The γ s165-181 peptide from ovine milk presents antimicrobial activity against various bacteria in food products like minced beef and UHT cream [123]. On this matter, CDPs’ natural antioxidant activity in meat processing is also promising as an alternative to synthetic antioxidants, which are often scrutinized for potential health risks [141,142]. While primarily studied in beef and poultry, these peptides could potentially be applied to other meat products, enhancing their quality and shelf life [142,143].

CDAMPs also play a natural and crucial role in fermented foods, providing a source of antimicrobial agents that inhibit spoilage and pathogenic microorganisms, contributing to product safety and extending shelf life [140]. Caseicidin and isracidin, naturally produced during casein hydrolysis, have been explored for their potential to inhibit pathogenic bacteria in dairy-based functional foods [144,145,146]. CDPs produced by *Lactobacillus acidophilus* fermentation have shown potential in bioprotective applications, particularly in milk-based formulas to prevent infections like those caused by *Cronobacter sakazakii* [76]. Whey protein films incorporating casein peptides have also been used to improve the microbial stability of refrigerated soft cheese, effectively inhibiting the growth of bacteria such as *E. coli* and *B. subtilis* [147].

#### 4.1.2. Food Packaging

The incorporation of CDPs into food packaging materials has also emerged as a promising strategy to enhance food preservation. In a broader sense, AMPs from different sources have been successfully integrated into films, coatings, and pouches, creating active packaging systems capable of extending the shelf life of perishable foods, and have been extensively reviewed elsewhere [148,149,150,151]. In the case of casein, its unique properties, especially high thermal stability, biodegradability, and micelle and emulsification forming capacity, as well as ability to bind metal ions, makes it an excellent biomaterial for edible coatings [20]. Active casein coatings and films can effectively extend the shelf life of perishable foods by hindering decay kinetics and incorporating bioactive compounds, such as phenolic acids, allowing a controlled release of antimicrobial agents into the food environment, reducing spoilage. β-casein, in particular, can be highly effective in carrying small molecules and ions due to its hydrophobic domain [152]. The interaction of bioactive additives with casein, and how they affect the structural properties of coatings and films, has been reviewed elsewhere [152]. Notably, the incorporation of phenolic acids and essential oils (e.g., cinnamon and lemongrass) enhanced the preservation of sensory and nutritional quality in fresh produce, even offering an alternative to nanoparticles and probiotics, while being biocompatible and biodegradable [147].

#### 4.1.3. Functional Foods

In the context of functional foods, all types of bioactive peptides can be added or enriched through modifications to standard manufacturing processes, such as altering process parameters or changing starter cultures. Some traditional foods have been redefined with innovative marketing strategies and this has become a growing market niche. The incorporation of CDPs into functional food formulations has been shown to improve gastrointestinal health, reduce inflammation, and enhance immune responses [20]. Furthermore, research has indicated that the fermentation process in dairy products can release these peptides, enhancing their bioactivity and health benefits [20]. Currently, CDPs can be incorporated into a range of functional foods, including protein-enriched beverages, sports nutrition products, and infant formulas [116]. For instance, bioactive peptides with antioxidant properties, such as casomorphins and lactoferricin-derived peptides, are being integrated into fortified dairy drinks and energy bars to enhance their health benefits [116]. Additionally, casein hydrolysates are increasingly used in health and medical nutrition [152] and can also provide a delivery system for antibacterial casein-derived peptides, such as caseicin A [153]. Examples of commercially available functional foods and ingredients containing casein-derived bioactive peptides are provided in Table 3. These applications have been a common trend in food markets for several years and have been reviewed [37,132]. All of them are aimed at casein’s health benefits, namely hypotensive, mineral absorption, and stress-reducing activities.

Of course, one of the most common approaches is the use of fermented dairy products such as yogurt, kefir, and cheese, where proteolytic enzymes from lactic acid bacteria generate CDPs with enhanced bioactivity. Several reports have shown the presence of CDPs in dairy products [154,155], and processed dairy products such as cheeses and fermented milk [34,51,155,156,157,158].

### 4.2. Pharmacological Applications

Beyond their established nutritional role, casein proteins from milk exhibit remarkable potential in pharmaceutical applications. Technological advancements in nano-delivery systems—such as liposomes, electrospun nanofibers, and polymeric nanoparticles [159,160,161]—have improved the protection of peptides from degradation and facilitated their controlled release in pharmaceutical, and cosmetic formulations. There are currently several pharmaceutical applications for casein-derived peptides that exhibit promising therapeutic effects in the management of hypertension, diabetes, and coagulation disorders [162,163,164], including innovative delivery methods such as casein-based hydrogels developed for the delivery and sustained release of biomolecules [165].

Regarding CDPs’ antimicrobial activities, they present several advantages as therapeutic alternatives: (1) they present rapid bactericidal activity; (2) they have broad-spectrum efficacy; (3) they act faster than bacterial replication rates, which may help prevent the development of antimicrobial resistance [37]; and (4) they are structurally suited to rapid absorption and targeted activity [166,167]. Moreover, CDPs have shown compatibility with conventional medications and generally exhibit minimal adverse effects [71].

Accordingly, there is a growing body of evidence that in both human and veterinary medicine, CDPs offer promising alternatives to traditional antibiotics, particularly in addressing antimicrobial resistance in livestock and antimicrobial resistant bacteria [136]. Kappacin, for instance, has shown significant antibacterial activity. A combination of kappacin and Zn2+ was demonstrated to suppress the in vitro growth of oral cariogenic bacteria, including biofilm formation, for extended periods [168]. Recent trials using human volunteers confirmed that a mouthrinse solution containing a glycomacropeptide (GMP)/kappacin formulation (1.33 mM) and zinc (ratio 1:15) exhibited comparable efficacy to chlorhexidine mouthrinses (0.05%) against dental plaque. This combination prompted the commercialization of a new product for treating oral diseases [169,170].

In the context of mastitis, a prevalent and costly disease in dairy cattle, CDPs such as isracidin have been found to exhibit strong antimicrobial properties. In a study, intramammary infusion of isracidin showed a significant reduction in somatic cell count, effectively curing mastitis in 90% of infected cows, caused by *S. aureus* and other bacteria that were resistant to conventional antibiotics [80]. In a similar acute mastitis model, the intramammary injection of isracidin (two doses of 2 g each) successfully treated 71% of udder quarters in cows previously infected with resistant *S. aureus* [80].

Furthermore, CDPs have shown promise in treating systemic infections. In a study, a single intramuscular injection of isracidin effectively protected mice against subcutaneous infections caused by *S. aureus*, *Streptococcus pyogenes*, *and Listeria monocytogenes* [80]. This study demonstrated that isracidin provided protection for at least five months, and treated mice survived a second *S. aureus* 8126 infection in the fifth month, unlike the control mice, where only around 10% survived the first infection. The protective effect was also observed in other animal models, including rabbits, guinea pigs, and sheep. In these models, isracidin was injected before infection with *S. aureus* strains, showing its effectiveness in preventing infection [80].

These findings support the potential of CDPs as alternatives to antibiotics, particularly in veterinary medicine, where resistance to conventional antibiotics remains a significant challenge.

### 4.3. Isolation, Stability, and Delivery Systems

Despite several health benefits and functionalities, consumption of bioactive peptides derived from casein is often limited by their undesirable flavor or bitter taste, low solubility, poor stability during processing, and poor oral bioavailability [159]. A key challenge is to enhance their bioavailability from natural sources or to formulate novel food products through the addition and/or fortification of isolated or concentrated peptide fractions.

Casein production in CDP isolation relies on manipulating the physicochemical properties of casein micelles, colloidal particles that precipitate at their isoelectric point (pH 4.6) [171]. Acid coagulation is a common method, destabilizing micelles and causing casein precipitation through calcium and phosphate removal [172]. Enzymatic coagulation, involving chymosin-mediated cleavage of κ-casein, generates gel-like structures under optimal conditions of pH 5.1–5.3 [173,174]. Coagulation can also be influenced by external factors such as pressure and CaCl_2_ addition [175]. Heat coagulation (130–150 °C) is another technique, with micelle size and milk composition affecting coagulation efficiency [176,177]. Microfiltration, utilizing 0.1 μm membranes, allows for the concentration of micellar casein, though efficiency can be impacted by membrane fouling and salt addition [133,178]. A more recent approach, high-pressure CO_2_ coagulation, preserves micelle stability by maintaining near-neutral pH and enhances rheological properties [179,180]. Chromatographic methods such as reverse-phase and ion-exchange chromatography are used for casein fractionation, with efficiency dependent on column type and mobile phase composition [181,182].

The functional properties of casein, including solubility and moisture-binding capacity, are critical for its applications. Casein solubility increases with pH adjustments and enzymatic hydrolysis [183], while its water-binding capacity is enhanced by the micellar structure and can be further increased through enzymatic treatments [183,184].

A significant challenge in harnessing the antibacterial potential of CDPs lies in their stability during digestion and food processing. These peptides, often sensitive to enzymatic degradation, can lose their antimicrobial activity when exposed to the acidic environment of the stomach or digestive enzymes. On this matter, the use of casein hydrolysate was shown to improve the bioavailability and antioxidant efficacy of CDPs, with acidic peptide fractions having higher bioavailability and resistance to digestive enzymes and intestinal peptidases [185]. Additionally, food processing conditions, such as heat or high pressure, may compromise the integrity of these peptides, diminishing their effectiveness. It has been reported that controlling enzyme/substrate ratio and hydrolysis time of bioactive peptides derived from casein hydrolysates can enhance both production and efficacy of CDAMPs [114]. A total of 70 unique peptides were identified using different enzyme/substrate ratio (E/S) and hydrolysis times, predominantly from αs1-casein. The authors showed that an increase in E/S ratio and hydrolysis time led to a decrease in peptide number. In silico analyses indicated that all peptides were non-toxic and non-allergenic, with several displaying high bioactivity potential according to PeptideRanker scores. These findings highlight the relevance of production conditions in tailoring the release of functional peptides, supporting their potential application in the functional food industry.

A growing number of studies have explored various delivery systems, such as micro and nano-encapsulation, nanocarriers, and hydrogels, to optimize and safeguard the release and preservation of peptides [186,187,188,189,190,191]. CDPs incorporated into nanostructured delivery systems have been successfully used to target gastrointestinal pathogens. A study by Wenck et al. (2024) [192] reported casein-coated and drug-loaded magnetic nanoparticles for theranostic applications. Also, casein peptide-loaded nanocarriers for enhanced antibacterial activity could be of use, as suggested by Lombardi et al. (2024) [193]. In food systems, delivery technologies such as edible films or emulsions have also been investigated for the incorporation of CDPs [194,195]. These systems not only protect the peptides from environmental factors but also offer controlled release, ensuring their effectiveness over time. For example, casein-derived antimicrobial peptides incorporated into food coatings have been shown to extend the shelf life of perishable products by inhibiting bacterial growth, without affecting food quality [138,196].

For clinical approaches, self-assembling peptide-based hydrogels are a promising offer regarding biocompatibility, biodegradability, and spatiotemporal control for precise wound healing and tissue repair [197]. Topical antimicrobial peptide formulations with high stability are also a growing trend, which could incorporate CDPs [198]. Oral mucosal casein salt films show good compatibility and desired drug release with no side effects, offering a biodegradable and effective drug delivery system for anti-diabetic drugs [199]. More advanced technologies such as nanoencapsulation of CDPs within electrospun nanofibers have also emerged [159].

Interestingly, despite the challenges in delivering CDPs, several studies suggest that casein-based formulations are themselves an excellent delivery system for controlled release systems, with applications in drug delivery for both hydrophilic and hydrophobic drugs, offering sustained release, improved bioavailability, and biocompatibility [200,201,202,203,204,205].

### 4.4. Safety and Regulatory Aspects of Casein-Derived Bioactive Peptides

Casein hydrolysates and specific peptides such as Val-Pro-Pro and Ile-Pro-Pro have been extensively evaluated for safety, showing no evidence of target organ toxicity or adverse effects in animal studies. Studies by Maeno et al. (2005) and Mizuno et al. (2005) reported no lowest observed effect level (LOEL) or maximum tolerated dose (MTD) below 2 g/kg/day in rats [206,207]. Long-term administration also showed no negative reproductive or developmental effects [208]. Tensguard^®^, a functional ingredient containing Ile-Pro-Pro, was found to be non-mutagenic and exhibited a no observed adverse effect level (NOAEL) equivalent to 2 g/kg/day [209].

Several bioactive peptides derived from casein, such as lactotripeptides (e.g., IPP and VPP), have demonstrated beneficial effects, including antimicrobial activity and blood pressure regulation. While specific casein-derived peptides have shown promising health benefits, regulatory approvals typically apply to casein hydrolysates as ingredients in food formulations. These peptides, often present in hydrolysates, are recognized for their bioactive properties but are not yet individually regulated for use in food products.

In terms of regulation, casein and caseinates are covered under EU regulations (EC 2921/90; EC 760/2008), but casein hydrolysates do not currently possess GRAS status or approved health claims in Europe (EFSA, 2009a). Therefore, they are subject to safety evaluations by regulatory authorities such as the European Food Safety Authority (EFSA). Recently, the EFSA allowed hydrolysates like Peptigen^®^ IF-3080 in infant formulas. Such hydrolysates are expected to be formalized in the EU regulations (Regulation (EU) 2016/17). Similar regulatory frameworks are in place in other regions, including Australia, New Zealand (FSANZ), and the United States (FDA), where casein hydrolysates are permitted in food products, provided they meet safety standards. Other specific casein-derived peptides such as β-casomorphin-7 (BCM-7) have been evaluated and found to pose no health risk (EFSA, 2009b). Iron milk caseinate was also authorized as a novel food in the EU in 2023 (EU) (2023/949). Specifically, its use is permitted in food for special medical purposes, certain foods, and food supplements, subject to certain conditions.

Conversely, Japan has developed a legal framework (FOSHU) for the use of functional foods since 1991, allowing scientific validation of health claims. Under this system, peptides like Val-Pro-Pro, Ile-Pro-Pro, Val-Tyr [210], and casein phosphopeptides (CPPs) [211] have been approved for anti-hypertensive and mineral bioavailability applications.

To ensure both efficacy and safety, validated biomarkers are required to assess the physiological impact of these peptides, alongside toxicological assessments to ensure absence of cytotoxicity, allergenicity, and other adverse effects [114,212,213,214].

## 5. Conclusions and Future Perspectives

Milk proteins, particularly casein, are among the most thoroughly studied precursors of bioactive peptides. Recent advances continue to unveil novel biological activities and novel amino acid sequences. The growing number of identified casein-derived peptides reflects increasing scientific interest in their multifunctional potential. These peptides exhibit unique structural and functional properties, with well-documented antimicrobial activity among other bioactivities. Their applications are expanding across the food sector—as natural preservatives, functional ingredients, and components in active packaging—as well as in pharmacological contexts, where they are emerging as viable and effective alternatives to conventional antibiotics, including evidence of in vivo efficacy. Despite challenges related to their inherent instability and sensitivity, particularly in delivery systems, recent advances have introduced promising strategies to ensure safe, efficient, and targeted delivery of these peptides. The growing interest in functional foods has led to the incorporation of several casein-derived peptides into commercial food products. This progress has been largely enabled by developments in large-scale processing technologies, such as membrane separation, ultrafiltration, and nanofiltration, which facilitate the isolation and concentration of specific peptides. Moreover, encapsulation strategies—including micro- and nanoencapsulation—offer innovative solutions to improve peptide stability during food processing and gastrointestinal digestion, thus enhancing their functional efficacy in complex food matrices.

From a regulatory standpoint, the progressive acceptance of casein hydrolysates and bioactive peptides highlights a positive trend toward their integration into food and health-related applications, but a lot remains to be accomplished. A key research priority is the accurate quantification and optimization of the bioavailability of bioactive peptides in different food systems. Finally, the incorporation of bioactive peptides into food products must be accompanied by safety evaluations and compliance with regulatory frameworks. This is particularly relevant when health claims are proposed, since regulatory bodies require robust scientific substantiation to approve such claims. Addressing these regulatory aspects will be crucial for the broader acceptance and commercialization of CDP-enriched functional foods and therapeutics.

## Figures and Tables

**Table 1:** Examples of CDPs: structures, bioactivities, and applications.

Category	Peptide Name	Sequence	Bioactivity	Concentration	Applications	References
1. Casomorphins	β-Casomorphin-5; β-Casomorphin-7	YPFPGPIPNSL; YPFPGPI	Opioid, ACE-inhibitory, immunomodulatory (µM)	10.00	Potential influence on immune and neurological systems; excessive intake may lead to gastrointestinal discomfort or altered immune responses.	[41,42]
2. Casokinins	Casokinin-10	YQQPVLGPVR	ACE-inhibitory (µM)	300.00	Potential applications in functional foods to manage hypertension, as a natural alternative to ACE inhibitors.	[43,44]
3. Lactoferricin-Like Peptides	Lactoferricin-like peptides derived from β-casein	FKCRRWQWRMKKLGAPSITCVRRAF	Antimicrobial (MIC) mg/mL	*B. cereus*—400; *B. subtilis*—6.3; *L. innocua*—6.3; *L. monocytogenes*—6.3; *E. coli*—12,5; *S. entiritidis*—25; *S. thyphimurium*—6.3	Natural antimicrobials for food safety and potential therapeutic agents in animal health.	[45,46]
4. αs1-Casein Hexapeptide	α-casozepine	YLGYLEQLLR	Anxiolytic		Used in stress relief products, studied for calming effects on stress-related behaviors.	[47,48]

**Table 2:** Antimicrobial peptides derived from casein: origin, sequence, target microorganisms, type of assay, and reported activity. NA—not available. Adapted from [31,36].

Peptide	Name	Sequence	Type of Assay	Bacteria Inhibition	References
Alpha-S1-Casein		YLEQLLR	MIC (μg/mL)	*B. subtilis*—53.6; *E. coli* NEB 5α—241.0; *E. coli* ATCC 25922—40.2	[75]
Alpha-S1-Casein	Caseicin B	VLNENLLR	MIC (mM); well diffusion assay	(1) *E. coli*—0.22; *C. sakazakii +++*; *L. innocua ++*; *L. bulgaricus ++*; *S. mutans ++*,	[76]
			MIC (mg/mL)	(2) *C. sakazakii*—0.97	[77]
			MIC (mM)	(3) *C. sakazakii*—1.25; *C. muytjensii*—0.625	[78]
Alpha-S1-Casein		TTMPLW	MIC (μM)	*E. coli*—193; *S. aureus*—193.0; *M. luteus*—64.0; *C. albicans*—144.0	[79]
Alpha-S1-Casein	Caseicin C	SDIPNPIGSENSEK	MIC (mM)	*L. innocua*—1.0	[76]
Alpha-S1-Casein		RPKHPIKHQGLPQEVLNENLLRFFVAPFPEVFGKEKV	MIC (μg/mL)	*B. subtilis*—29.0; *E. coli* NEB 5α—58.0; *E. coli* ATCC 25922—58.0	[75]
Alpha-S1-Casein		RPKHPIKHQGLPQEVLNENLLRFFVAPFPEVFGKEK	MIC (μg/mL)	*B. subtilis*—59.0; *E. coli* NEB 5α—59.0; *E. coli* ATCC 25922—59.0	[75]
Alpha-S1-Casein		RPKHPIKHQGLPQEVLNENLLRFFVAPFPEVFGK	MIC (μg/mL)	*B. subtilis*—31.4; *E. coli* NEB 5α—31.4; *E. coli* ATCC 25922—31.4	[75]
Alpha-S1-Casein		RPKHPIKHQGLPQEVLNENLLRFF	MIC (μg/mL)	*B. subtilis*—23.6; *E. coli* NEB 5α—47.2; *E. coli* ATCC 25922—189.0	[75]
Alpha-S1-Casein	Isracidin	RPKHPIKHQGLPQEVLNENLLRF	MBC (mg/mL)	*E. coli*—0.1—1.0;	[80]
			MIC (mg/mL)	*C. sakazakii*—0.5; *E. coli*—0.2	[81]
Alpha-S1-Casein		RPKHPIK	MIC (μg/mL)	*Lactobacillus sakei* A15—200.0; *Escherichia coli* K12—400.0; *Bacillus megaterium* F6—400.0	[82]
Alpha-S1-Casein	CP1/Cpep11	LRLKKYKVPQL	MIC (μM)	(1) *E. coli* ATCC 25922—64.0; *E. coli* UB1005—128.0; *S. pullorum*—256.0; *Salmonella enterica subsp enterica* CMCC 50071—256.0; *S. aureus* ATCC 29213—640.0, *L. monocytogenes*—65.0	[9]
			MIC (μg/mL)	(2) *B. subtilis*—125; *L. innocua*—125.0; *L. monocytogenes* FSAW 2310—125.0; *L. monocytogenes* NCTC 11994—125.0; *C. freundii*—500; *E. aerogenes*—>1000; *E. coli*—250.0; *S. enteritidis*—250.0; *S. thyphimurium*—125.0	[83]
			MIC (μg/mL)	(3) *S. dysenteriae* ATCC51302—300.0; *E. coli* ATCC 25922—250.0; *Salmonella enterica* serovar Typhimurium ATCC 14028—125.0; *B. subtilis* ATCC 9372—275.0; *S.aureus* ATCC25923—125.0; *Streptococcus pneumoniae* ATCC 49619—150.0	[84]
Alpha-S1-Casein		LGYLEQLLRL	MIC (μg/mL)	*B. subtilis*—NA; *E. coli* NEB 5α—NA; *E. coli* ATCC 25922—1356.0	[75]
Alpha-S1-Casein		LEQLLRLKKY	MIC (μg/mL)	*B. subtilis*—1266.0; *E. coli* NEB 5α—633.0; *E. coli* ATCC 25922—NA	[75]
Alpha-S1-Casein	Fragment of Isradicin	IKHQGLPQEV	MIC (μg/mL) and MBC (μg/mL)	*E. coli*—MIC 25.0–50.0, MBC 50.0; *B. subtilis* MIC 50.0, MBC 100.0	[85]
Alpha-S1-Casein	Caseicin A	IKHQGLPQE	MIC (mg/mL)	(1) *E. coli*—2.0; *L. monocytogenes*—1.0; *S. thyphimurium*—2.0; *P. putida*—1.0; *S. aureus*—0.5	[71]
			MIC (mM)	(2) *E. coli* DPC6053—0.05	[76]
			MIC (mM)	(3) *C. sakazakii*—0.625; *C. muytjensii*—0.625; *S. thyphimurium*—1.25; *E. coli*—1.25; *K. pneumoniae*—1.25; *P. fluorescens*—1.25	[78]
Alpha-S1-Casein		HIQKEDVPSERYLGYLEQLLRLKKYK	MIC (μg/mL)	*B. subtilis*—42.4; *E. coli* NEB 5α—169.0; *E. coli* ATCC 25922—NA	[75]
Alpha-S1-Casein		HIQKEDVPSERYLGYLEQLLRLKK	MIC (μg/mL)	*B. subtilis*—186.0; *E. coli* NEB 5α—NA; *E. coli* ATCC 25922—NA	[75]
Alpha-S1-Casein		HIQKEDVPSERYLGYLEQLLRLK	MIC (μg/mL)	*B. subtilis*—292.0; *E. coli* NEB 5α—292.0; *E. coli* ATCC 25922—584.0	[75]
Alpha-S2-Casein		PYVRYL	log(N0/Nf)	*E. coli*—0.27; *S. carnosus*—2.23; *S. epidermis*—2.06; *L. innocua*—1.13	[86]
Alpha-S2-Casein		WIQPKTKVIPYVRYL	MIC (μM)	*C. sakazakii*—78.125; *L. monocytogenes*—39.063	[87]
Alpha-S2-Casein	Casein F/CP2	VYQHQKAMKPWIQPKTKVIPYVRYL	MIC (mM)	(1) *B. subtilis*—21; *L. innocua*—21; *L. monocytogenes*—21; *C. freundii*—664; *E. coli*—332; *S. enteritidis*—664; *S. typhmirium*—21,	[83]
			MIC (μM)	(2) *C. sakazakii*—312.5 *L. monocytogenes*—78.125	[87]
			MIC (μM)	(3) *E. coli*—16.0; *L. innocua*—16.0; *B. cereus*—16.0; *M. flavus*—16.0; *St. thermophilus*—8.0	[88]
			MIC (μg/mL)	(4) *E. coli*—8.0–16.0; *S. carnosus*—8.0–16.0,	[89]
			MIC (μM)	(5) *E. coli*—1.25; *S. choleraesuis*—0.5; *S. Epidermidis*—2.5; *L. monocytogenes*—0.05	[90]
Alpha-S2-Casein		TVYQHQKAMKPWIQPKTKVIPYVRYL	MIC (μg/mL)	*B. subtilis*—2.7; *E. coli* NEB 5α—21.4; *E. coli* ATCC 25922—172.0	[75]
Alpha-S2-Casein		TKVIPYVRYL	MIC (μM)	*C. sakazakii*—156.25 *L. monocytogenes*—78.125	[87]
Alpha-S2-Casein		TKLTEEEKNRLNFLKKISQRYQKFALPQYLK	MIC (μg/mL)	*B. subtilis*—4.0; *E. coli* NEB 5α—16.2; *E. coli* ATCC 25922—16.2	[75]
Alpha-S2-Casein	P14	TKKTKLTEEEKNRL	MIC (μM)	*B. cereus*—2.99; *S. aureus*—2.3; *L. monocytogenes*—2.99	[91]
Alpha-S2-Casein	CR7	QKFALPQYLKTVYQHQKAMKPWIQPKTKVIPYVRYL	MIC (μg/mL)	*B. subtilis*—312.5; *L. innocua*—625.0	[46]
Alpha-S2-Casein	P10	QKALNEINQF	MIC (μM)	*B. cereus* 0.87; *S. aureus*—1.75; *L. monocytogenes* 1.75; *H. pylori*—0.083	[91]
Alpha-S2-Casein	CR3	LKTVYQHQKAMKPWIQPKTKVIPYVRYL	MIC (μg/mL)	*B. subtilis*—312.5; *L. innocua*—625.0	[46]
Alpha-S2-Casein	CR5/CR6	LKKISQRYQKFALPQYLKTVYQHQKAMKPWIQPKTKVIPYVRYL	MIC (μg/mL)	*B. subtilis*—4.8; *L. innocua*—4.8; *L. monocytogenes* 2310—4.8	[46]
Alpha-S2-Casein		LKKISQRYQKFALPQY	MIC (μM)	*E. coli*—25.0; *L. innocua*—50.0; *B. cereus*—75.0; *M. flavus*—75.0; *St. thermophilus*—50.0	[88]
Alpha-S2-Casein	CR1	KTVYQHQKAMKPWIQPKTKVIPYVRYL	MIC (μg/mL)	*B. subtilis*—21.0; *L. innocua*—21.0; *L. monocytogenes 2310*—21.0; *S. typhimirium*—21.0	[46]
Alpha-S2-Casein		KTKLTEEEKNRLNFLKKISQRYQKFALPQYLKTVYQHQK	Diffusion Assay	*E. coli*—NA; *S. carnosus*—NA	[92]
Alpha-S2-Casein	SR4	KKISQRYQKFALPQYLKTVYQHQK	MIC (μg/mL) and MBC (μg/mL)	*E. coli*—MIC 50.0, MBC 100.0; *B. subtilis* MIC 12.5–25.0, MBC 50.0	[85]
Alpha-S2-Casein		KAMKPWIQPKTKVIPYVRYL	MIC (μM)	*C. sakazakii*—39.063; *L. monocytogenes*—39.063	[87]
Alpha-S2-Casein		KAMKPWIQPKTKVIP	MIC (μM)	*C. sakazakii*—>1.250; *L. monocytogenes*—156.25	[87]
Alpha-S2-Casein	SR9	KAMKPW	MIC (μg/mL) and MBC (μg/mL)	*E. coli*—MIC 150.0, MBC 300.0; *B. subtilis* MIC 150.0, MBC 600.0	[85]
Alpha-S2-Casein		IVLNPWDQVK	MIC (μg/mL)	*B. subtilis*—1363.0; *E. coli* NEB 5α—681.0; *E. coli* ATCC 25922—1363.0	[75]
Alpha-S2-Casein	SR1	IQPKTKVIPYVR	MIC (μg/mL) and MBC (μg/mL)	*E. coli*—MIC 50.0, MBC > 100.0; *B. subtilis* MIC 50.0, MBC > 100.0	[85]
Alpha-S2-Casein	CR4	ALPQYLKTVYQHQKAMKPWIQPKTKVIPYVRYL	MIC (μg/mL)	*B. subtilis*—10.7; *L. innocua*—10.7; *L. monocytogenes* 2310—10.7; *S. typhimirium*—21.4	[46]
Alpha-S2-Casein		SSSEESII	MIC (mM)	*L. innocua* ATCC 33090—0.205; *Micrococcus luteus* ATCC 4698—0.818; *E. coli* ATCC 25922—0.654; *S. enteritidis* ATCC13076—1.145	[93]
Alpha-S2-Casein		KTVDMESTEVFTKKTKLTEEEKNRLNFLKK	MIC (mM)	*B. subtilis* ATCC6633—3.10–6.67	[94]
Beta-Casein		YPVEPF	Diffusion assay	*Bacillus* spp.—NA; *L. monocytogenes*—NA	[95]
Beta-Casein	Bc3	EMPFPK	MIC (μg/mL)	*E. coli* PTCC 1399—60.0; *S. aureus* PTCC 1431—25.0	[96]
Beta-Casein	Casecidin 17	YQEPVLGPVRGPFPIIV	MIC (mg/mL)	*E. coli* DH5a—0.5; *E. coli* DPC6053—0.4	[81]
Beta-Casein	Casecidin 15	YQEPVLGPVRGPFPI	MIC (mg/mL)	*E. coli* DH5a—0.5; *E. coli* DPC6053—0.4	[81]
Beta-Casein	Bc6	VLPVPQK	MIC (μg/mL)	*E. coli* PTCC 1399—45.0; *S. aureus* PTCC 1431—25.0	[96]
Beta-Casein	Bc8	VKEAMAPK	MIC (μg/mL)	*E. coli* PTCC 1399—30.0; *S. aureus* PTCC 1431—10.0	[96]
Beta-Casein	SR8/Bc5	AVPYPQR	MIC (μg/mL) and MBC (μg/mL)	(1) *E. coli*—MIC 100.0, MBC > 100.0; *B. subtilis* MIC 50.0, MBC > 100.0	[85]
			MIC (μg/mL)	(2) *E. coli* PTCC 1399—40.0; *S. aureus* PTCC 1431—20.0	[96]
Beta-Casein		VPYPQRDMPIQAFL	MIC (μg/mL)	*E. coli* NEB 5α—493.0	[75]
Beta-Casein	Bc14	VLPVPQKAVPYPQR	MIC (μg/mL)	*E. coli* PTCC 1399—30.0; *S. aureus* PTCC 1431—10.0–15.0	[96]
Beta-Casein		RINKK	MIC (mM)	*E. coli* 2.59	[97]
Beta-Casein	Bc11	HKEMPFPK	MIC (μg/mL)	*E. coli* PTCC 1399—50.0; *S. aureus* PTCC 1431—20.0	[96]
Beta-Casein	Bc12	EAMAPKHK	MIC (μg/mL)	*E. coli* PTCC 1399—30.0; *S. aureus* PTCC 1431—15.0	[96]
Beta-Casein	Bc1	EAMAPK	MIC (μg/mL)	*E. coli* PTCC 1399—60.0; *S. aureus* PTCC 1431—25.0	[96]
Beta-Casein	LGDT2	VAGTWY	log(N0/Nf)	*B. subtilis*—2.2	[98]
Beta-Casein	LGDT1	IPAVFK	log(N0/Nf)	(1) *B. subtilis*—2.2; *S. zooepidemicus*—0.4	[98]
			MIC (μg/mL)	(2) *E. coli*—55.0; *S. aureus*—30.0	[99]
Beta-Casein		IDALNENK	log(N0/Nf)	(1) *L. monocytogenes*—0.72; *S. aureus*—1.03; *E. coli*—0.63	[99]
			log(N0/Nf)	(2) *E. coli*—70.0; *S. aureus*—35.0	[100]
Beta-Casein	LGDT3	VLVLDTDYK	log(N0/Nf)	*B. subtilis*—2.4	[98]
Beta-Casein		TPEVDDEALEK	log(N0/Nf)	*L. monocytogenes*—1.03; *S. aureus*—1.23; *E. coli*—0.69	[100]
Beta-Casein		IRL	log(N0/Nf)	*L. ivanovii*—NA; *E. coli*—NA	[101]
Beta-Casein	LGDT4	AASDISLLDAQSAPLR	log(N0/Nf)	*B. subtilis*—1.5; *S. lentus*—1.5; *S. zooepidemicus*—0.6	[98]
Kappa-Casein	Kappacin	MAIPPKKNQDKTEIPTINTIASGEPTSTPTTEAVESTVATLEDSPEVIESPPEINTVQVTSTAV	MIC (mg/mL)	(1) *E. faecalis*—0.64	[102]
			MIC (μg/mL)	(2) *S. mutans*—59.0	[103]
Kappa-Casein		YVL	log(N0/Nf)	*E. coli*—> 6; *S. maracescens*—3.08; *L. innocua*—> 6.0; *S. carnosus* > 6.0	[97]
Kappa-Casein		IQY	log(N0/Nf)	*E. coli*—> 6.0; *S. maracescens*—0.39; *L. innocua*—0.27; *S. carnosus*—0.22	[97]
Kappa-Casein		YYQQKPVA	log(N0/Nf)	*E. coli*—3.46; *S. maracescens*—0.04; *L. innocua*—0.66; *S. carnosus*—1.14	[97]
Kappa-Casein		VQVTSTAV	log(N0/Nf)	*E. coli*—0.10; *S. maracescens*—0.25; *L. innocua*—1.99; *S. carnosus*—1.17	[97]
Kappa-Casein		VESTVATL	log(N0/Nf)	*E. coli*—>6; *S. maracescens*—0.59; *L. innocua*—1.89; *S. carnosus*—3.44	[97]
Kappa-Casein	SR13	TEAVESTVATL	MIC (μg/mL)	*E. coli*—50.0; *B. subtilis*—50.0	[85]
Kappa-Casein		STVATL	log(N0/Nf)	*E. coli*—0.67; *L. innocua*—0.42; *S. carnosus*—0.29	[97]
Kappa-Casein		PAAVRSPAQILQ	log(N0/Nf)	*E. coli*—>6.0; *S. maracescens*—0.20; *L. innocua*—0.84; *S. carnosus*—0.97	[97]
Kappa-Casein		MMK	MIC (μg/mL)	*E. coli*—125.0; *S. aureus*—70.0	[104]
Kappa-Casein		MAIPPKKNQDKTEIPTINT	MIC (mg/mL)	*E. coli*—0.25	[105]
Kappa-Casein		IAK	MIC (μg/mL)	*E. coli*—100.0; *S. aureus*—60.0; *L. casei*—70.0; *L. acidophilus*—70.0	[103]
Kappa-Casein		FSDKIAK	log(N0/Nf)	*E. coli*—>6.0; *S. maracescens*—0.27; *L. innocua*—>6.0; *S. carnosus*—>6.0	[97]
Kappa-Casein		FFSDK	MIC (μg/mL)	*E. coli*—200; *S. aureus*—90	[104]
Kappa-Casein		EIPT	log(N0/Nf)	*E. coli*—2.59; *S. maracescens*—1.69; *L. innocua*—0.88; *S. carnosus*—0.74	[97]
Kappa-Casein		AVESTVATLEDSPEVIESPPE	MIC (μg/mL)	*S. mutans*—59.0	[103]

MIC: Minimum Inhibitory Concentration; MBC: Minimum bactericidal concentration.

**Table 3:** Examples of commercially available functional foods or food ingredients containing casein-derived bioactive peptides. Adapted from [37,132].

Product Name	Manufacturer	Type of Food	Peptide Name
Calpico (Europe) or Calpis AMEAL S (Japan)	Calpis Co., Japan	Fermented milk	B ioactive peptides Ile-Pro-Pro (IPP) and Val-Pro-Pro (VPP) from b- and k-CN
Capolac	Arla Foods, Denmark	Ingredient	Casein phosphopeptides
Casein DP Peptio Drink	Kanebo, Japan	Soft drink	Casein-derived dodecapeptide FFVAPFPEVFGK
CE90CPP	DMV, Netherlands	Ingredient	Casein phosphopeptides (CPPs)
C12 Peptide	DMV, Netherlands	Ingredient	Casein-derived dodecapeptide FFVAPFPEVFGK
Evolus	Valio, Finland	Fermented milk, calcium enriched	C12
Kotsu Kotsu calcium	Asahi, Japan	Soft drink	CPP
PeptoPro	DSM Food Specialists, Netherlands	Ingredient	Hydrolyzed casein
ProDiet F200	Ingredia, France	Milk Drink, Confectionary	CPP
Tekkotsu Inryou	Suntory, Japan	Soft drink	CPP
Peptigen^®^ IF-3080	Arla Foods, Denmark	Infant formulas	Casein hydrolizates

## Data Availability

Not applicable.
